# A Novel Resistive Switching Identification Method through Relaxation Characteristics for Sneak-path-constrained Selectorless RRAM application

**DOI:** 10.1038/s41598-019-48932-5

**Published:** 2019-08-27

**Authors:** Ying-Chen Chen, Chao-Cheng Lin, Szu-Tung Hu, Chih-Yang Lin, Burt Fowler, Jack Lee

**Affiliations:** 1 Microelectronics Research Center, Department of Electrical and Computer Engineering, The University of Texas at Austin, Austin, TX 78758 USA; 2 Taiwan Semiconductor Research Institute, TSRI, Hsinchu, Taiwan; 3 Material Science and Engineering Program, The University of Texas at Austin, Austin, TX 78712 USA; 4 Department of Physics, National Sun Yat-Sen University, Kaohsiung, Taiwan

**Keywords:** Electrical and electronic engineering, Electronic devices

## Abstract

Resistive random access memory (RRAM) is a leading candidate in the race towards emerging nonvolatile memory technologies. The sneak path current (SPC) problem is one of the main difficulties in crossbar memory configurations. RRAM devices with desirable properties such as a selectorless, 1R-only architecture with self-rectifying behavior are potential SPC solutions. In this work, the intrinsic nonlinear (NL) characteristics and relaxation characteristics of bilayer high-k/low-k stacked RRAMs are presented. The intrinsic nonlinearity reliability of bilayer selectorless 1R-only RRAM without additional switches has been studied for their ability to effectively suppress SPC in RRAM arrays. The relaxation properties with resistive switching identification method by utilizing the activation energy (Ea) extraction methodology is demonstrated, which provides insights and design guidance for non-uniform bilayer selectorless 1R-only RRAM array applications.

## Introduction

In recent years, memory technology includes static random access memory (SRAM), dynamic random access memory (DRAM), flash memory are encountering challenges due to the continued scaling down of the designs^1–4^. Among several types of next generation memory devices, resistive random access memory (RRAM) composed of a simple metal-insulator-metal (MIM) structure has increasingly been attracting much attention as a promising candidate for next-generation nonvolatile emerging memory according to its potentially ultra-high density production probability, faster switching speed (<10 ns), compatibility with a crossbar structure with CMOS integration, lower energy consumption, and the feasibility for neuromorphic computing architecture design^5–10^.

The RRAM with MIM structure is simplifying memory array design by crossbar architecture, however, the leakage through the sneak paths inevitably induced while accessing this RRAM crossbar networks. The sneak paths current (SPC) problem is one of the major issues in the development of three-dimensional (3D) crossbar memory design. The SPC problem can be described as the leakage from neighboring unselected cells (USC), which significantly results in the cross-talk and distorts the data of selected cell (SC) during reading operation. To mitigate the sneak paths currents, a diode or a selector device series with a RRAM cell to form 1D-1R or 1S-1R structure has been developed^11–15^. Several solutions on selection devices including Mott transition switches, nonlinear volatile switches, threshold switches, rectifying diode devices etc. have been presented^16–20^. Unfortunately, the additional selection devices for 1S-1R configurations considerably increase fabrication process, circuit design complexity, and additional cost per chip. Therefore, a selectorless memory composed of 1R-only design architecture with nonlinear characteristics is desirable for high-density RRAM array applications.

In our previous work, we reported the selectorless RRAM in high-k/low-k bilayer stacks, in which the intrinsic nonlinearity has been demonstrated by inserting a low-k layer (e.g. SiO_x_ layer or graphite oxide layer) and optimized by SET compliance current limit (CCL) modulation^21–24^. In addition, the bilayer or multilayer nonuniform metal-oxide-stacked structures for self-rectifying behavior have been studied, e.g. TiO_x_/HfO_x_, TaO_x_/TiO_x_, Al_2_O_3_/TiO_x_, WO_3_/WO_x_ etc.^25–34^. However, the mechanism of the nonlinearity-CCL responses, and reliability characteristics are not yet been investigated. This work not only studied the reliability of relaxation characteristics under temperature variation, but also proposed a switching identification method which provides the potential guidance for future design of 3D sneak-path-constrained selectorless crossbar RRAM configurations.

## Fabrication Process

The starting substrates were heavily-doped N + Si wafers. Titanium nitride (TiN) of 200 nm was deposited as the bottom electrode (BE). Then, 9 nm of SiO_x_ followed by 4 nm of HfO_x_ were deposited as resistive switching dielectric layers for realizing the bilayer selectorless structures by radio frequency (RF) sputtering method^10–13^. After switching layers deposition, 165 nm platinum was then deposited as top electrode (TE), as followed by lift-off method for RRAM devices. The SiO_x_ (9 nm) single layer devices, HfO_x_ (11 nm) single layer devices, HfO_x_ (7 nm)/graphite (5 nm), SiO_x_ (7 nm)/graphite (5 nm) are used as references. Graphite is deposited by the RF sputtering method as followed by the oxide layers and top electrode. For simplifying the device notifications, the abbreviation of HfO_x_ as “H”, SiO_x_ as “S”, and graphite as “G” are used following by the thickness of thin films. An Agilent B1500 and Lakeshore probe station were used for electrical characterization of the RRAM devices.

## Results and Discussion

**Figure 1:**
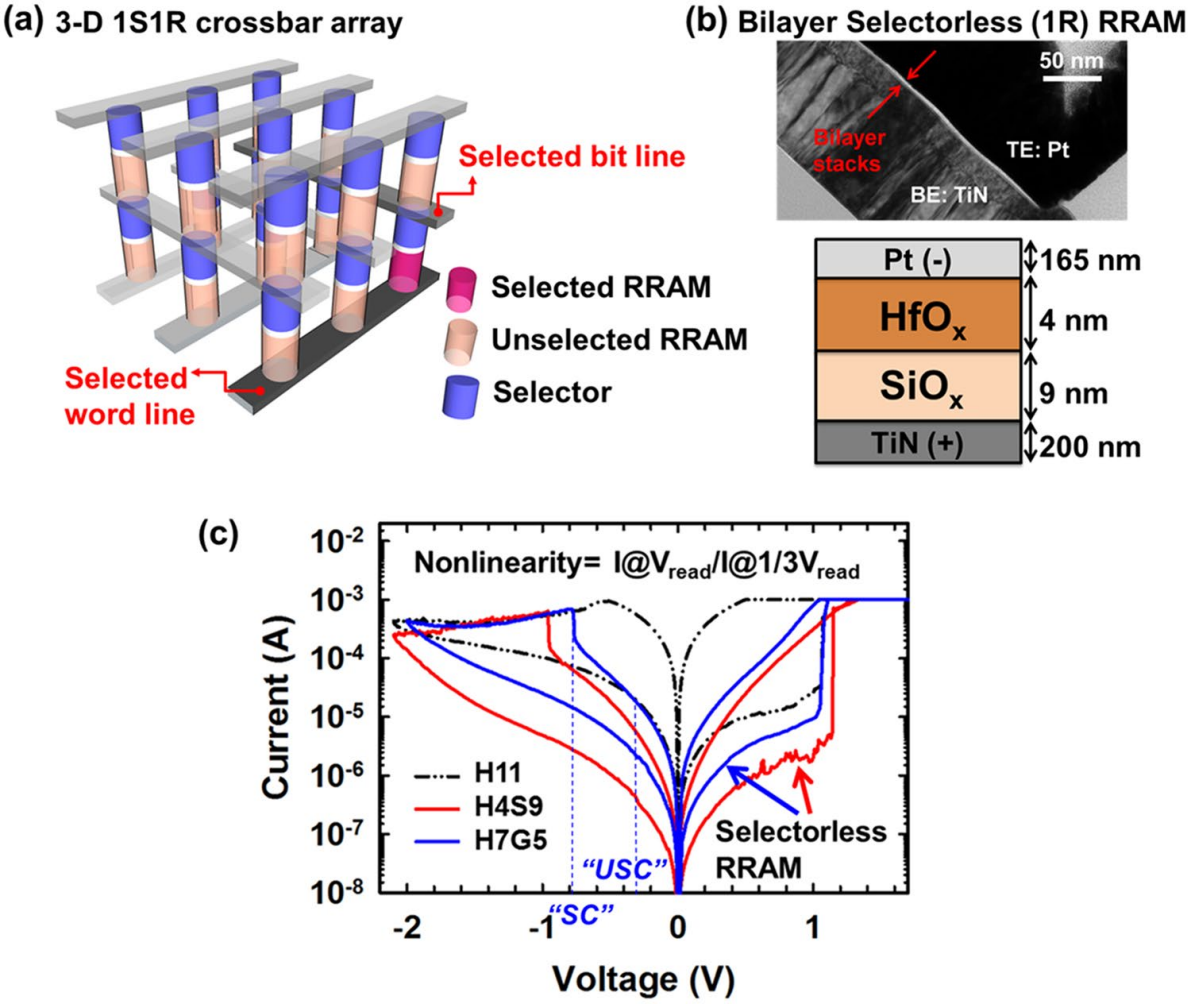
(**a**) Schematics of 1S1R crossbar array configuration for high class storage memory, (**b**) bilayer engineering structure design for selectorless RRAM with intrinsic nonlinearity in array applications, (**c**) I–V characteristics of HfO_x_ RRAM (H11), 1R selectorless RRAM of HfO_x_ (4 nm)/SiO_x_ (9 nm) (H4S9) and HfO_x_ (7 nm)/graphite (5 nm) (H7G5).

The schematic of 3D crossbar 1S-1R array memory configuration is shown in Fig. 1(a). Figure 1(b) shows the transmission electron microscopy (TEM) image of bilayer HfO_x_/SiO_x_ stacked device. The TEM sample is prepared by focused ion beam milling method with the scanning electron microscope (SEM). To initiate the resistive switching, a single voltage sweep electroforming process with a current limit was applied to induce a soft breakdown. After electroforming, the device manifests an improved conductance as the conductive filament (CF) connects the TE and BE, thus resulting in a low-resistance state (LRS) of the RRAM. The reset process can then be applied to rupture the CF, resulting in a high-resistance state (HRS). Then, the soft-breakdown process was performed by single sweeping the voltage until current abruptly increased to a compliance current limit (CCL) of 1 mA, as shown in Fig. 1(a). Voltage was applied to the bottom electrode (TiN) with the top electrode (Pt) connected to ground. By switching set and reset operation, the CF can be repeatedly connected/ruptured, and allowing reversible transition cycles between HRS and LRS. The SET process i.e. switching from HRS to LRS took place in positive polarity, while the RESET occurred in negative polarity.

Figure 1(c) shows bipolar resistive switching I-V characteristics during DC voltage sweeps for single HfO_x_ layer (H11) and bilayer selectorless RRAMs (H4S9 and H7G5). The I-V characteristics of H11 (black dash) and H4S9 (red) have been shown in our previous work^22^. In this experiment, the total thickness of bilayer and single layer devices are designed to be ~11 to 13 nm, in order to reduce the influence of bias stress i.e. overset during the electroformation process. The extra bias stress though forming process with potentially different shape of filaments is being avoided in here. After the electroforming process, the resistive switching performance was stabilized by 30 DC voltage sweep cycles. The SET process for oxide-based RRAMs was performed by applying to 3 V forward/reverse double sweep with 1 mA CCL to program to LRS. The RESET process was done by sweeping up to −2.1 V, where current decreased as the voltage was swept from around RESET voltage, and the devices were then programmed into HRS. The nonlinear nature in selectorless RRAM is shown to mitigate the SPC because the LRS of selected cell can be read at a “high-voltage” region (i.e. −0.8 V), while the sharp conductance drops at “low-voltage region” (i.e. −0.4 V or −0.28 V) effectively suppresses SPC through unselected cells in reading schemes (e.g. V/2 or V/3 read schemes)^35,36^. In other words, the sneak paths current can be constrained by leverage the nonlinearity of the self-rectifying current-voltage characteristics.

The nonlinearity is defined as the current at the read voltage (V_read_) divided by the current at the one third read voltage (1/3 V_read_) with V/3 scheme (half of read voltage (1/2 V_read_) with V/2 scheme). The on-state of the selected cell (SC) is read at a “high-voltage” (i.e. V_read_) region, while the sharp conductance decreases at “low-voltage region” (i.e. 1/3 V_read_ or 1/2 V_read_) effectively suppresses the sneak current through the unselected cells (USC). The nonlinearity of H7G5 (NL~120) stacked device exhibits ~24 times of improvement over that of H11 device (NL~5) with SET CCL of 1 mA, which suggests a significant increased current at V_read_ by inserting the high-k layer i.e. HfO_x_ in bilayer devices, with reduced current at 1/3 V_read_ (~10^−5^ A) for both structures. The high-k layer i.e. HfO_x_ in H7G5 is shown to enhance the higher currents than those of H4S9 are around −0.8 V, where the same high-k layer and same SET CCL of 1 mA (i.e. switching gap in low-k layer) are utilized.

**Figure 2:**
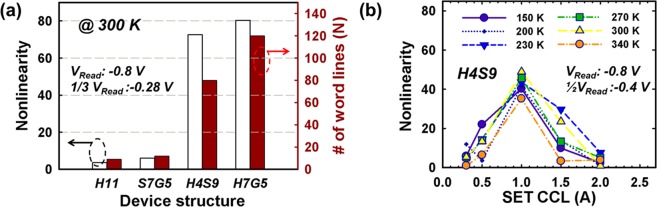
(**a**) Nonlinearity and calculated number of word lines (N) in various structures, (**b**) temperature effect of nonlinearity with SET CCL modulation on H4S9.

The early failure yields are as 62.5%, 37.5%, and 7.7% for H7G5, S7G5, H4S9, respectively, which depicts the H4S9 as having better DC cycling endurance than graphite-based selectorless RRAMs^37^. The number of word lines (N) assessment with nonlinearity by utilizing the V/3 read schemes are showed in Fig. 2(a), for single layer and bilayer selectorless RRAMs (median of 30 devices). The reading voltages are −0.8 V for fully read on selected cell, and −0.28 V for unselected cells. The bilayer devices i.e. H4S9 (or H7G5) have the nonlinearity of ~14 times (or ~18 times) higher than single layer device i.e. H11. After the calculation of array size by taking into account 10% read margin, the number of word line (i.e. the maximum array size) are 80 for H4S9 and 120 for H7G5, respectively. Although the H4S9 has slightly lower nonlinearity than H7G5, the early failure yield is also lower in H4S9 than in H7G5. In other words, there is a tradeoff between the reliability of memory window with the nonlinearity^37^.

In addition, the nonlinearity readouts under various temperature conditions with SET compliance currents limit (CCL) modulation is showed in the Fig. 2(b). The results show the nonlinearity properties are not affected by the ambient temperature under vacuum (~2.5 mtorr). The SET CCL modulation is applied under room temperature of 300 K, and 20 cycles for switching stabilization of each CCL condition are applied on devices. After DC cycles, the ambient temperature decreases from 300 K to 150 K, and the nonlinearity is characterized by V/2 scheme after the target temperature is reached for 5 minutes. The temperature elevation (i.e. 340 K, orange curve) is also applied and the nonlinearity shows slightly decrements comparing to the cooling process, which is thought to be suggested that the thermal effect on filamentary structures decreases the bandgap and increase the effective dielectric constant resulting in nonlinearity decrement^38^.

**Figure 3:**
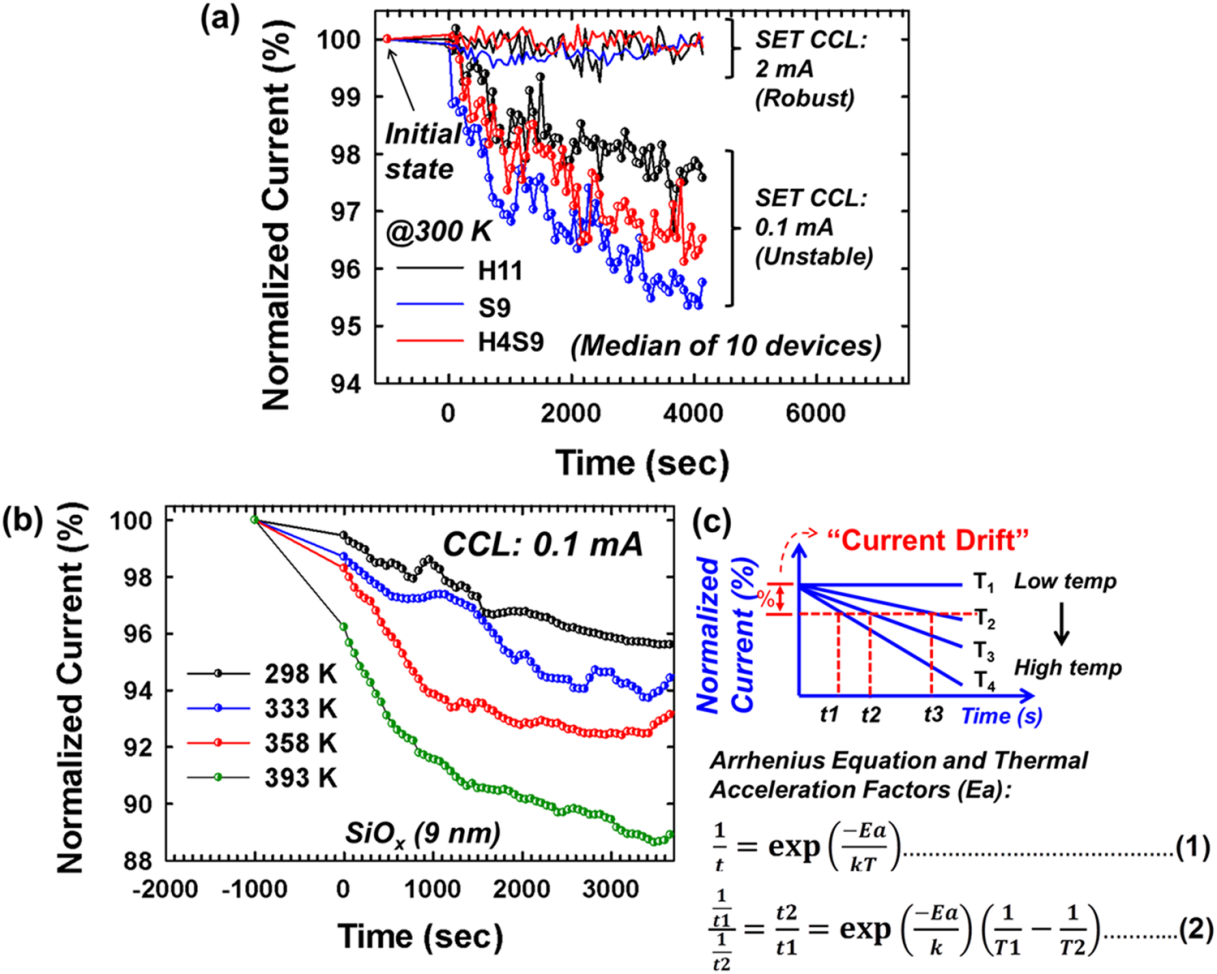
(**a**) The normalized current (%) change during filament relaxation with SET CCL of 0.1 mA and 2 mA under room temperature (black: H11, blue: S9, red: H4S9), (**b**) current reduction with temperature modulation of SiO_x_ (9 nm), (**c**) activation energy (E_a_) extraction methodology by relaxation behavior under various temperature conditions.

The relaxation characteristics of conductive filament with varied SET CCL of 0.1 mA and 2 mA for H11, S9, H4S9 (median of 10 tested devices for each structure) are compared and showed in Fig. 3(a). The normalized current (%) is defined as the current of time (I_t_) divided by initial current (I_0_) multiply by 100%. The current drift is the difference between two current percentages, i.e. I_0_-I_t_/I_0_. Here, the current drift of 5% is chosen as a criterion to extract the activation energy. In other word, the time value utilized for E_a_ extraction is as I_t_ have 5% of current drift (i.e. normalized current is 95%). The retention testing is applied every 60 seconds, and read voltage is 0.1 V. The results showed the current drift is larger as the SET CCL is lower in all the device structures, e.g. current drift ~5% with CCL of 0.1 mA while <1% with CCL of 2 mA on S9 after 4000 seconds. This is thought to be suggested that the conductive filamentary structures are thicker with higher SET CCL (e.g. 2 mA), which results in better retention and less current drift^39^. On the other hand, the H11 showed the greater relaxation behavior (current drift ~2.5%) in the comparison of H4S9 (current drift ~3.8%) and S9 (current drift ~5%).

Figure 3(b) shows the current drift as a function of time on S9 devices under various temperate conditions. With increasing temperature, the larger the filamentary structures relaxation occurs, i.e. ~11% under 393 K, ~8% under 358 K, ~5% under 333 K, ~4% under 298 K after 1 hour. Based on the observation of different relaxation behaviors with temperature on various devices, the methodology of switching identification is proposed (Fig. 3(c)). The Arrhenius equation and extracted activation energy (E_a_) are utilized, where the t is relaxation time under 5% current drift, T is the ambient temperature (in kelvin) during retention testing, k is the gas constant of 8.314 × 10^−3^ kJ mol^−1^K^−1^. By comparing the extracted activation energy value as an indicator, the information of filamentary structure composition and resistive switching can possibly be identified, which will be discussed in next session.

**Figure 4:**
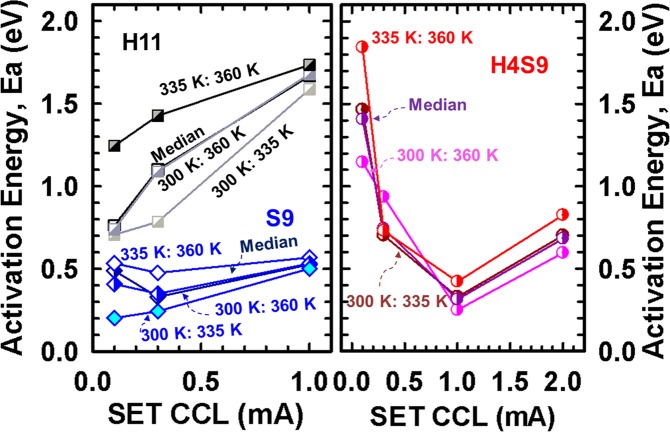
Activation energy (E_a_) extraction methodology by relaxation behavior under various temperature conditions (black curve for H11; blue curve for S9; red curve for H4S9).

The extracted Ea values as a function of SET CCL with various temperatures are shown in Fig. 4 (black curve for H11; blue curve for S9; red curve for H4S9). The temperature of 300, 335, 360 K have been used in the retention measurements and relaxation behavior characterizations. The extracted activation energy (E_a_) values based on Arrhenius equation are in the range of ~0.7 to 1.8 eV for single layer HfO_x_, and ~0.3 to 0.4 eV for single layer SiO_x_ (Fig. 4, left panel). The extracted Ea values for H4S9 bilayer selectorless devices with SET CCL modulation is showed in Fig. 4 (right panel). The preliminary result shows the nonlinearity characteristics of H4 and H11 with SET CCL of 1 mA are 3.08 and 3.04, respectively^22–24^. The nonlinearity is independent on the thickness of HfOx single layer devices, so the H11 as the reference sample to avoid extra voltage stress. Noted the Ea of HfO_x_ (4 nm) is of ~1.87 eV at CCL of 1 mA, and not showing significant differences than HfOx (11 nm) (~1.67 eV). The extracted Ea value of H4S9 bilayer devices is in the median of ~0.32 eV as CCL is of 1 mA, and ~1.46 eV, ~0.7 eV, ~0.7 eV as CCL are of 0.1, 0.3, and 2 mA, which suggested that the resistive switching at SET CCL of 1 mA has Si and O ionized defects involved in the filament structures than other CCL conditions. The analysis of RESET process (i.e. filament rupture process) is the based on the “hourglass model” as well as quantum point contact (QPC)^40,41^ model to present the oxygen vacancies or metal ions movements during switching process. The relaxation behavior of filament utilized here for Ea extraction is also analyzed based on the hourglass model, where the thinnest part of conductive filament i.e. bottle neck is only composed several metal atoms. During the relaxation process, the conductance of CF continues to decrease until fully ruptured the CF, where the metal filament dissolution process determines the process i.e. M-M bonds continue to break which requires less bond dissociation energy than M-O formation^42,43^. When the last atom is dissolved, the conductive filament is finally ruptured to HRS. Besides, the relaxation of Si-CF is faster than Hf-CF (Fig. 3(a)) which corresponds to the bond energy of Si-Si (~3.2 eV) is lower than Hf-Hf (~4.02 eV), while the bond energy of Si-O (8.15~8.42 eV) is similar to which of Hf-O (8.16~8.43 eV)^42,43^. In other words, the Si metal filamentary structure is comparably weaker than Hf metal filamentary structures which have higher Ea and lower reaction rate for LRS relaxation. The bond energy of Si-Si bond is ~20% lower than of Hf-Hf bond, which explains the lower extracted Ea value showed in the SiO_x_ single layer devices. According to Figs 2(b) and 4 (right panel), the H4S9 with SET CCL of 1 mA is showing the higher nonlinearity related to the Si filamentary structure than other CCL conditions, which depicts the optimized nonlinearity can be achieved by both modulating the CCL and insertion of a low dielectric constant layer.

## Conclusion

In conclusion, the intrinsic nonlinearity has been demonstrated in bilayer selectorless 1R-only RRAM without additional diode/transistor selector elements, which are beneficial in suppressing SPC in the high-storage-class crossbar memory array configuration. The resistive switching identification method utilizing reliability of relaxation properties, SET CCL modulation, and activation energy extraction have been reported, where the E_a_ is ~0.7 to 1.8 eV for single layer HfO_x_, ~0.3 to 0.4 eV for single layer SiO_x_, respectively. The relaxation characteristics and resistive switching identification provide the insights and mechanism understanding of bilayer selectorless 1R-only RRAM for high storage class crossbar memory configuration.
